# An Overview on *Rumex dentatus* L.: Its Functions as a Source of Nutrient and Health-Promoting Plant

**DOI:** 10.1155/2022/8649119

**Published:** 2022-07-22

**Authors:** Atif Ali Khan Khalil, Falak Zeb, Raees Khan, Sayed Afzal Shah, Esra Küpeli Akkol, Ishaq N. Khan, Jawad Khan, Syed Babar Jamal, Fazli Khuda, Adnan Haider, Saeed Ahmed, Naveed Ur Rehman

**Affiliations:** ^1^Department of Biological Sciences, National University of Medical Sciences, Rawalpindi 46000, Pakistan; ^2^Department of Clinical Nutrition and Dietetics, Research Institute for Medical and Health Sciences, University of Sharjah, Sharjah, UAE; ^3^Department of Pharmacognosy, Faculty of Pharmacy, Gazi University, Etiler 06330, Ankara, Turkey; ^4^PK-NeuroOncology Research Group, Institute of Basic Mdical Sciences, Khyber Medical University, Peshawar 25000, Pakistan; ^5^Department of Pharmacy, University of Peshawar, Peshawar 25120, Khyber Pakhtunkhwa, Pakistan; ^6^College of Pharmacy and Research Institute of Pharmaceutical Sciences, Gyeongsang National University, Jinju 52828, Republic of Korea

## Abstract

*Rumex dentatus* L. (Polygonaceae), also known as toothed dock or Aegean dock, is a medicinal plant with a high culinary value in addition to being used as an ethnomedicinal plant. This review focuses on the botanical, nutritional, phytochemical, and pharmacological activities of *R. dentatus*, as well as the future prospects for systematic investigations into these areas. *R. dentatus* has been subjected to scientific evaluation, which has confirmed its traditional uses and demonstrated a wide range of biological and pharmacological potentials, including antioxidant, anticancer, antifungal, antibacterial, anti-inflammatory, and other biological properties. Phytochemical analyses showed the presence of anthraquinones, chromones, flavonoids, and essential oils. As a result of this current review, the medicinal significance of *R. dentatus* has been confirmed, and future research on its unexplored aspects, such as the identification of pharmacologically active chemical constituents and related mechanisms and safety, may be stimulated, with the goal of developing it into a drug.

## 1. Introduction

In earlier times, humans relied on a wide variety of secondary metabolites and natural products (NPs) for the synthesis of medicines in order to treat a wide range of diseases. NPs are typically obtained through the extraction process from plants, animals, and even occasionally from microbes [1]. In general, it has been stated that natural products possess a number of pharmacological properties, and it is utilised in a number of different therapeutic approaches [2]. According to the findings of Jaradat et al. [3], it was found that 80 percent of the world's population relies on the use of NPs for their healthcare requirements [3, 4]. In addition, it has been demonstrated that natural products (NPs) play an essential role in the development and discovery of new drugs and are capable of warding off a wide range of diseases. This is demonstrated by the fact that 25% of all drugs used in clinical practice are NP replacements [5].

As was discussed in the previous section, these NPs exhibit a variety of pharmacological properties such as anti-inflammatory [6], antibacterial [7], antihelminthic [8], antioxidant [9], antiviral [10], anticancer [11], and antifungal properties [12]. In addition, compounds derived from natural sources that are known for their anticancer effects include vincristine, vinblastine, paclitaxel, and camptothecin, as well as their derivatives [13].

A total of more than 150 species of plants are found in the genus *Rumex*, which is extensively distributed around the world. According to some research, quinones, especially anthraquinone, and flavonoids are the major secondary metabolites of this species [14]. It has a large number of medicinally significant plant species that are beneficial in the treatment of a variety of serious disorders [15]. Among them, *R. dentatus* is a medicinally important plant and is distributed globally. The roots of this species have been displayed to contain a number of significant bioactive compounds such as quercetin, kaempferol, myricetin, chlorogenic acid, and vitamin C [16]. This plant has been reported to have anticancer, antibacterial, antifungal, anti-inflammatory activities, etc. [16]. However, there does not appear to be a complete compilation of scientific data on the nutritional, phytochemical, and pharmacological profiling of *R. dentatus*. The purpose of the current review is to provide more detail regarding the pharmacological and biological importance of *R. dentatus* in order to assist researchers in comprehending the role that this substance plays in the process of drug discovery and to encourage further investigation.

## 2. Diagnostic Features, Habitat, and Global Distribution

*R. dentatus,* belonging to the flowering plant (Polygonaceae), can grow up to 70 cm, is a tall herb that is usually unbranched or sometimes branched from the base. Smaller stem leaves and 3–4 toothed valves are its distinguishing characteristics. However, it is a highly polymorphic species; it is represented by several (7) geographical races from South East Europe across North Africa and the warmer parts of Asia [17], that are mostly known taxonomically as subspecies [17]. A taxon native mostly to East and South Asia such as India, Japan, China, and Korea and typically recognized as *R. dentatus* subsp. *klotzschianus*. Furthermore, *R. dentatus* subsp. *halacsyi* (*R.* *×* *halacsyi*) is native to the Caucasus, East Mediterranean region, South and East Europe, and parts of Central and Southwest Asia.

**Figure 1:**
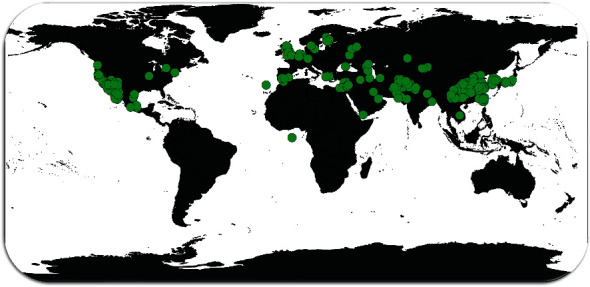
*Rumex dentatus* global distribution. The occurrence records from the GBIF were used to create the following map: GBIF.org (22 June 2021) GBIF Occurrence Download https://doi.org/10.15468/dl.gexb3e.

*R. dentatus* generally occurs in moist valleys, mountain slopes, or plains to an elevation of 3000 m [18]. Its native range is Tunisia to Indo-China. However, it is also introduced to parts of Western Africa, Western Europe, and North America. The global distribution shows that it occurs predominantly in the subtropical to temperate regions of the Northern Hemisphere (Figure 1).

## 3. Ethnobotany

The leaves of *R. dentatus* are refrigerant, diuretic, and utilised as a cooling agent [19] and astringent [20]. The root of *R. dentatus* is used in cutaneous disorders [21]. It is used as a vegetable and as a purgative [22].

*R. dentatus* has also been reported to have toxic effects on humans causing dysentery and gastric pains [23]. It is also used as an appetizer and cholagogue [24]. Several other studies reported that *R. dentatus* is used against ailments of the integumentary system, gastrointestinal disorders, especially diarrhea, in wound healing and as an antidote, and skin allergy. The fresh root of *R. dentatus* is chewed for some time which helps to cure the tooth gum swelling [25]. Farooq et al. reported that the root extract of *R. dentatus* is used against constipation [26]. Two spoons of the root extract are mixed with tea and taken daily for fifteen days. Other studies have reported that *R. dentatus* is traditionally used for its antitumor, antidermatitis, anti-inflammatory, tonic, laxative, diuretic, astringent, and bactericidal properties [27–29]. Ajaib et al. have reported that the leaves of *R. dentatus* are used as carminative, diuretic, and stomachic [30].

## 4. Nutritional Composition

**Figure 2:**
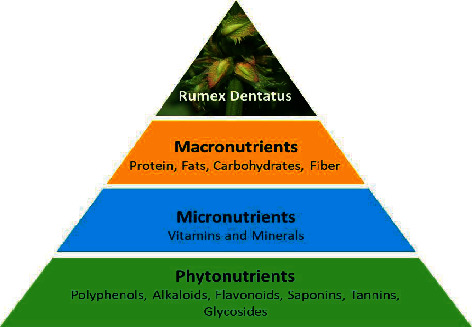
Nutritional composition of *R. dentatus*.

Plants have been used as therapeutic agents since the beginning of time, both in unorganized and organized forms [31]. *R. dentatus* is one of these plants that is widely recognized for its essential biochemical and nutritional position because it contains a reasonable amount of fat, fibres, carbohydrates, proteins, minerals, and vitamins. *R. dentatus* is also recognized for producing a significant amount of ethanol [32, 33]; dynamic sources of minerals and vitamins always played an important role in times of food shortage and are also used as traditional remedies for many ailments [34]. A study in Pakistan analyzed the nutritional value of different parts of the *R. dentatus* plant and showed that the percentage of protein was highest in the stem (15.72) followed by the flower (1.76), leaf (13.75), seed (12.12), and fruit (10.50); the percentage of fat was highest in the root (14.66) followed by the flower (13.00) and leaf (12.50); the percentage of fiber was highest in the fruit (11.67) followed by the flower (10.88) and root (10.65); the percentage of carbohydrates was highest in the seeds (54.40) followed by the fruit (52.84) and leaf (52.05) [35]. A recent study also demonstrated that *R. dentatus* contains fiber (12.40%), fats (2.83%), protein (11.95%), carbohydrates (56.37%), and calories per 100 g (298.75 kcal). However, the mineral composition was carbon (52.02%), oxygen (32.75%), Mg (0.53%), potassium (8.00%), calcium (0.21%), and iron (0.25%) [36]. In addition, this plant also possesses phytochemicals which have a favorable impact on human health [37]. Phytochemicals such as proteins, glycosides, carbohydrates, flavonoids, alkaloids, tannins, phenols, saponins, and steroids have been shown to have healing effects as well as to have physiological activity in the body. Preliminary phytochemical studies showed that *R. dentatus* contains saponins, tannins, terpenoids, flavonoids, cardiac glycosides, and alkaloids. Furthermore, syringic acid, vanillin, cinnamic acid, benzoic acid, ferulic acid [36, 38, 39], chlorogenic acid, myricetin, quercetin, vitamin C, and kaempferol were detected by HPLC [16]. This composition indicates that *R. dentatus*. Has a high nutritive value in terms of macronutrients, micronutrients, and phytonutrients that are essential for a healthy lifespan (Figure 2).

## 5. Phytochemistry

**Figure 3:**
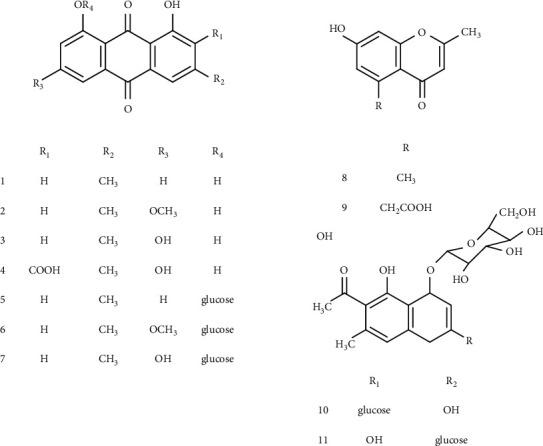
Chemical structures of quinones, chromones, and naphthalene glucosides.

**Figure 4:**
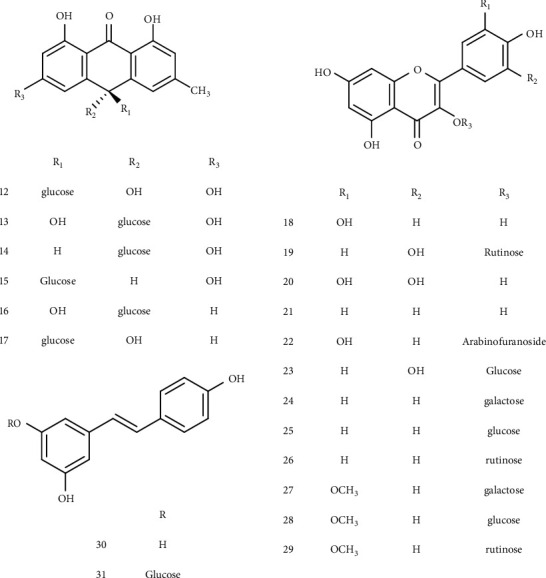
Chemical structures of C-glucosyl anthrones, flavonoids, and stilbenes.

**Figure 5:**
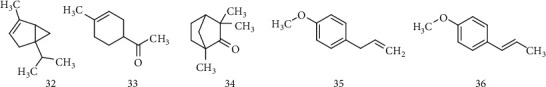
Chemical structures of monoterpenes.

**Figure 6:**
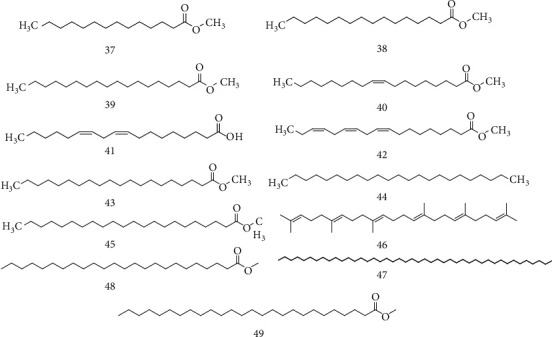
Chemical structures of fatty acid methyl esters.

**Figure 7:**
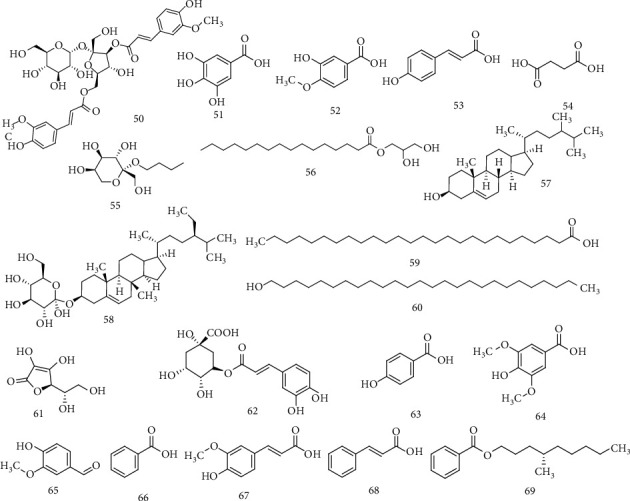
Chemical structures of other compounds.

Preliminary phytochemical studies revealed that *R. dentatus* contains quinones, flavonoids, terpenoids, cardiac glycosides, alkaloids tannins, and saponins [38]. A total of 63 compounds have been isolated and identified. These compounds included quinones, chromones, naphthalene glucoside, c-glucosyl anthrones, flavonoids, stilbenes, and essential oils. In this section, we will go through the key chemical ingredients of this plant, their structures, and the portions of the plant that are used for isolation (Table 1; Figures 3–7).

The *Rumex* genus is distinguished by the presence of a significant proportion of anthraquinone derivatives. A colorimetrical analysis revealed that *R. dentatus* contained 0.485% of anthraquinone derivatives, which was even higher than the quantity found in other species of *Rheum* [14]. *R. dentatus* roots were examined, and seven different anthraquinones were found. Among them, physcion, chrysophanol, emodin, endocrocin, physcion-8-O-*β*-D-glucopyranoside, emodin-8-O-*β*-D-glucopyranoside, and chrysophanol-8-O-*β*-D-glucopyranoside were identified as major chemical constituents in this plant (Figure 3) [40].

Two chromones (2-methyl-5-carboxymethyl-7-hydroxychromone and 7-hydroxy-2,5-dimethylchromone) and two naphthalene glucosides (6-methyl-7-acetyl-1,8-dihydroxy naphthalene-1-O-*β*-D(L)-glucoside and 6-methyl-7-acetyl-1,8-dihydroxy-3-methoxynaphthalene-1-O-*β*-D(L)-glucoside) have been isolated from this plant till now (Figure 3) [40, 41, 51].

Six C-glucosyl anthrones were identified as rumejaposide E, rumejaposide F, rumejaposide G, rumejaposide H, cassialoin, and rumejaposide from the roots of *R. dentatus* (Figure 4) [42].

Till now, a total of six flavonoids have been isolated from this plant. These flavonoids include quercetin, avicularin, quercitrin, rutin, myricetin, and kaempferol (Figure 4) [16, 43, 44].

Plant stilbenes, which are synthesized through the general phenylpropanoid route, have only been detected in a few higher-degree plant species thus far [52]. This plant was shown to contain resveratrol and polydatin (Figure 4) [46].

There had been little research done on the volatile components of *R. dentatus*. According to one study, the essential oils found in the leaves of *R. dentatus* were extracted by steam distillation and solvent extraction, and then evaluated by GC/MS, yielding five distinct peaks that could be differentiated. The most important and major volatile chemicals were found in these peaks, and they were a-thujene, limonene, fenchone, estragole, anethole, etc. (Figure 5) [47].

Palmitic acid methyl ester, meristic acid methyl ester, 7-C16:1, 10-oleic acid methyl ester, stearic acid methyl ester, linoleic acid, linolenic acid methyl ester, C20:0 methyl ester, hydrocarbon C23H48, C22:0 methyl ester, squalin, hydrocarbon C44H90, C24:0 methyl ester, and C26:0 methyl ester were identified by GC-MS in *R. dentatus* (Figure 6) [47].

Ten more chemical constituents were identified and isolated from *R. dentatus*: isovanillic acid, succinic acid, gallic acid, *p*-hydroxycinnamic acid, quercetin, hexadecanoic acid 2, *n*-butyl-beta-D-fructopyranoside, 3-dihydroxy propyl ester, daucosterol, helonioside A, and *β*-sitosterol. Furthermore, chlorogenic acid, vitamin C, vitamin A, *p*-hydroxybenzoic acid, cinnamic acid, syringic acid, ferulic acid, and vanillin benzoic acid which were also detected by HPLC, fatty acid, and momoterpenes (Figure 7) [16, 48–50].

## 6. Biosynthesis of Anthraquinones and Chromones

**Figure 8:**
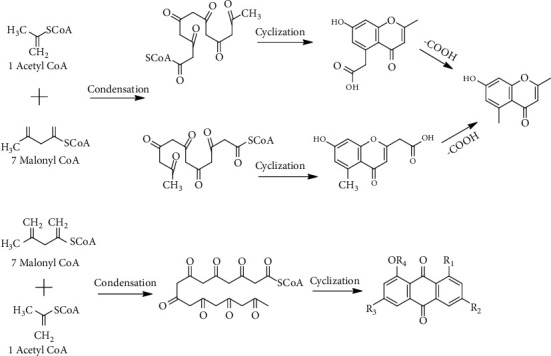
Biosynthesis of anthraquinones and chromones.

Secondary metabolites that were extracted from *R. dentatus* were quite comparable to those that are present in rhubarb; it is possible that a polyketide pathway based on acetate malonate for the biosynthesis of anthraquinones and chromones found in *R. dentatus* is identical to that found in rhubarbs (Figure 8) [53]. The C12 polyketide precursors are formed by the condensation of one molecule of acetyl- CoA with five molecules of malonyl-CoA, resulting in the formation of a polyhexanone, which can then be converted into various chromone intermediates containing either an acetoxyl at C-2 or C-5, depending on the polyketide precursor; then, by loss of the carboxyl, the final product, 7-hydroxy2,5-dimethylchromone, is finally obtained (Figure 8) [40]. A polyoctanone is formed when one acetyl-CoA molecule condenses with the molecules of seven malonyl-CoA, and an anthraquinone skeleton is formed after cyclization in the case of the C16 polyketide precursors (Figure 8) [53, 54].

## 7. Pharmacological Activities of *Rumex dentatus*

It is well recognized that *R. dentatus* possesses potentially substantial biological actions against particular kinds of cancer, reactive oxygen species, inflammations, microorganisms, neurotoxicants, and carcinogens. Multiple in vitro and in vivo studies have provided evidence that supports its pharmacological profile as well as its mechanistic effects as an anticancer drug.

### 7.1. Anticancer Activity

Cancer is the most difficult type of disease to treat, and it can have fatal consequences. There have been decades of enhanced research towards early cancer diagnosis, improving chemotherapeutic abilities, and improving prognostic results; nonetheless, this remains a difficult undertaking to complete because of the inherent molecular and genomic intricacies. Despite the fact that several scientific studies have been carried out to better understand the various components of cancer, no comprehensive and full solution has yet been established [55]. Dysregulated death of cells in tissues and unrestrained cell division are the hallmarks of cancer [56, 57]. By moving through blood and lymph, cancers can infiltrate neighboring tissues of the same organ as well as distinct organs throughout the body [57].

According to the International Agency for Research on Cancer (IARC) estimates, roughly 7.6 million people die from cancer every year across the world [58]. According to the International Agency for Research on Cancer (IARC) estimates, 17 million cancer cases were reported in 2018, with a total of 9.5 million cancer deaths reported worldwide. By 2040, it is anticipated that the worldwide cancer burden would reach 16.3 million deaths and 27.5 million new cancer cases, primarily as a result of population expansion and ageing [59]. More than half of all fatalities worldwide, in both industrialised and developing countries, are caused by cancer, and cancer progression rates are continuously growing [60, 61]. This increase can be attributed to a variety of factors, including industrialization and socio-economic growth, which have all had an impact on the overall quality of life and the prevalence of poor lifestyles [62]. The two most important factors are a decreased response to pharmacotherapy and a low therapeutic index [63].

**Figure 9:**
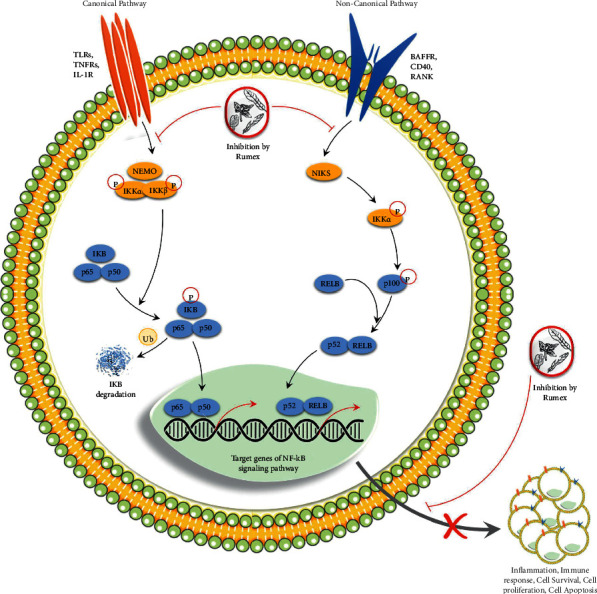
Anticancer effects of *R. dentatus* via molecular modulations of both the canonical and noncanonical pathways.

NPs derived from a variety of medicinal plants serve as an excellent source of bioactive molecules for research [64] because they have the potential to inhibit proliferation and have anticancer properties [56]. As a result, if done effectively, a complete examination of NPs could be a viable and practical solution for determining the NPs' medicinal potential with known dosage profiles. The use of NPs and their derivatives has made a realistic intervention to reduce cancer incidence conceivably, and this method is increasingly being more extensively adopted [65]. It has been demonstrated that several of these naturally occurring multitarget chemicals are efficient in regulating the growth patterns, differentiation, and proliferation rate of cancer cells [66]. According to the findings of certain researchers, NPs are favoured in clinical medicine due to the fact that they have a low toxicity level, a high safety rating, and are simple to obtain. By stimulating the production of antioxidant and detoxifying enzyme systems, they have the potential to halt, even reverse cancer progression [67]. The consumption of NPs in the diet can reduce the chance of developing cancer by twenty percent, as well as the mortality rate associated with cancer by a factor of twenty thousand deaths each year across the globe [67]. *R. dentatus* is an extremely diversified plant. The molecular activity of diverse chemicals isolated from this plant has been linked to its benefits in the treatment of many acute and chronic disorders, including malignancies, according to numerous studies. Cancer cells, such as malignant cells, breast cancer MCF-7, oophoroma SKOV-3, melanoma A375, and gastric cancer 7901, have been proven to be susceptible to *R. dentatus*' anticancer effects. The antiproliferative action of this plant was discovered to be associated with cell cycle arrest at the G0/G1 phase, as well as activation of apoptosis and accumulation in the sub-G1 phase, as revealed by the methanol and chloroform extracts of this plant. Moreover, Batool et al. confirmed that both methanolic and chloroform extracts prevented the proliferation of malignant cells and triggered their death by inhibiting the activation of nuclear factor-B and its following transcripts, Bcl-xL, Bcl-2, cyclin D1, survivin, and XIAP, as well as the expression of NF-B (Figure 9). The presence of caspase-3 in the cells confirmed the presence of apoptosis in the cells as well. Malignant cells were also rendered incapable of invasive and migrating due to the effects of methanolic and chloroform extracts, which also inhibited IBa phosphorylation in the tumour cells [68]. Furthermore, the antiproliferation activities were also performed by Zhang et al. for isolated compounds from this plant against breast cancer MCF-7, oophoroma SKOV-3, melanoma A375, and gastric cancer 7901. The compounds chrysophanol, 6-methyl-7-acetyl-1, 8-dihydroxy naphthalene-1-O-*β*-D(L)-glucoside and 6-methyl-7-acetyl-1, and 8-dihydroxy-3-methoxy naphthalene-1-O-*β*-D(L)-glucoside were identified to have anticancer effects [41]. In addition, several important major chemical constituents (emodin, physcion, physcion-8-O-*β*-D-glucopyranoside, chrysophanol, and chrysophanol-8-O-*β*-D-glucopyranoside) of this plant isolated from other related species were proven to have anticancer effects via multiple molecular signaling pathways such as apoptosis intrinsic pathway caspase-3, p53-dependent autophagy, cell cycle, PI3K-AKT, JAK-STAT, MAPK-ERK, MAPK-JNK/p38, Wnt, and immunomodulation (TNF-*α*- NF-*κ*B) pathways (Figure 9) [69–71]. Overall, it has been confirmed that this medicinally important plant has active secondary metabolites, which might be a rich source of anticancer drugs.

### 7.2. Antioxidant Activity

According to the World Health Organization, almost 80 percent of the world's population uses herbal traditional medicines for primary health care, with the bulk of these therapies containing plant extracts as active ingredients [72]. Several studies have shown that when our bodies are under stress, our bodies produce more reactive oxygen species (ROS) than tenzyme antioxidants. Superoxide anion radical (O_2_–), hydroperoxyl radical (HO_2_), and hydroxyl radical (OH) are among them, as are other non-free radicals such as H2O2 [73]. These free radicals, in the absence of antioxidants, aid in the development of a range of diseases, including cancer, diabetes, liver cirrhosis, atherosclerosis, cardiovascular disease, inflammation, and a variety of neurological disorders [38, 74]. Butylated hydroxy anisole (BHA) and butylated hydroxytoluene (BHT) are synthetic antioxidants that have been widely utilised in the food sector, but they have been linked to carcinogenesis and liver cirrhosis [73]. As a result, natural antioxidants have received a lot of attention in recent decades. Traditional medicines have been used to treat various disorders for a long time, and their scientific validation has led to the invention of a slew of new pharmaceuticals.

Globally, the *Rumex* species have been widely used in many traditional medical systems including Chinese, Ayurvedic, and African medicine. In China, a juice made from the roots has been used to treat bacterial and fungal infections such as enteritis, dysentery, ascariasis, diarrhea, and eczema [41, 42]. In India, the whole plant extract has been used as an astringent [44].

Regarding antioxidant activity, the whole plant has been tested using DPPH assay [38]. The ethanol extract showed maximum inhibition (96%) at 300 *µ*g/ml compared to ascorbic acid (95%). Likewise, the methanol extract showed 85% inhibition at the same dose. In addition, the butanol fraction showed 90% activity when tested against lipid peroxidation assay (Table 2) [75]. From the results, it is evident that *R. dentatus* possess highly significant antioxidant activity even comparable to that of the standard, and thus could be used as an antioxidant supplement.

### 7.3. Antibacterial Activity

Scientists are actively seeking novel active chemicals from natural resources in their search for new antimicrobials. In the past, phyto-remedies and edible oils were extensively utilised to treat a variety of infectious and noninfectious diseases [5]. There are various studies which have reported the effectiveness of extracts from *R. dentatus* against various pathogenic and nonpathogenic bacterial strains (Table 2). However, the exact compound responsible for such activities still awaits to be identified, except hexacosanol which was derived from the mentioned plant source and has been known to be effective against many microbial species (Table 2).

### 7.4. Antifungal Activity

The widespread problem of antibiotic resistance, along with the high burden and newly observed prevalence of invasive fungal infections, has compelled researchers to look for innovative medicines that have either no or little adverse effects. In recent years, there has been a significant increase in the number of researches that focus on NPs and herbal medicine [5]. Almost, a total of 100 NPs have been investigated in the last decade, including polyketides, terpenoids, alkaloids, and other peptides. Studies are limited on the antifungal efficacy of *R. dentatus*. However, few studies have reported that extracts or compounds from *R. dentatus* could potentially inhibit various pathogenic and nonpathogenic fungi growth (Table 2).

One practical example of the efficacy of *R. dentatus* against fungal pathogens is the use of this plant in traditional Chinese medicine preparation against Netherland dwarf fungal skin disease. This traditional Chinese medicine comprises the following components: *R. gmelinii* root, *R. dentatus* leaf, dried alum, *Cucurbita moschata* seed, *Litsea verticillata* root, *Portulaca oleracea*, *Firmiana platanifolia* seed, *Fallopia multiflora* root, *Panax ginseng* leaf, *Astragalus membranaceus* root and *Perilla frutescens* stem. The preparation method includes (1) breaking these components into powder of 70–150 mesh; (2) feeding the powder to the decoction container, adding water, soaking for 15–25 min, heating to boil, decocting with low fire for 40–60 min, separating decoction, decocting the dregs for 2–3 times, collecting decoction, and filtering through filter screen of 100–150 mesh to remove residue; (3) pouring the decoction to the decoction container, heating to 60–80°C, and evaporating at low temperature. This traditional Chinese medicine not only treats the Netherland dwarf fungal skin disease, but also has the functions of inhibiting bacteria and relieving itching, clearing away heat and toxic substances, strengthening the spleen and stomach, improving the immunity, and promoting the recovery of rabbit hair. More advantages include easy availability of components, scientific compatibility, simple manufacture, low cost, quick effect, no toxicity or side effects, no easy recurrence, and a high cure rate (>85%) [45, 49, 76].

### 7.5. Antiviral Activity

As a nasty particle wrapped up in a protective protein shell, a virus is what it sounds like [80]. Human life is thought to be at risk from viral infections, which are considered to be one of the most serious risks [81]. It is now common knowledge that NPs derived from a variety of natural sources constitute the single most important source of antiviral agents for the treatment of COVID-19 [82, 83]. Five different extracts were prepared using ethanol, methanol, benzene, n-hexane, and chloroform solvents. It was found that the methanol extract of *R. dentatus* had the greatest antiviral activity against DENV-2 replication, with IC50 values of 0.154 and 0.234 micrograms per milliliter of solution, respectively, when applied before infection with 45 and 90 PFU of virus, respectively (Table 2) [77].

### 7.6. Anti-Inflammatory Activity

Inflammation can be brought on by a number of different things, the most common of which are tissue injury or damage and infection. Carcinogenesis, autoimmune disorders, and cardiovascular diseases are all related with inflammation, and inflammation is often the result of these events as well [84]. When it comes to various circumstances, inflammatory responses can be induced by a chronic infection or by exposure to noxious substances that cause the inflammation to be activated. Both of these scenarios are examples of situations in which inflammation can be activated. Many different intracellular signaling pathways, such as transcription factors, kinases, and cell surface receptors, are often dysregulated in chronic inflammation. In a normal situation, inflammation kicks off the activation of a number of different protein kinases, including AKT/PI3K, MAPK, and JAK, in addition to the families of protein kinases associated with the members of these protein kinase families, with the intention of modifying the progression of metastasis [85]. Several *Rumex* species have been reported to have anti-inflammatory activities such as *R. vesicarius*, *R. nepalensis*, and *R. patientia* [86–88]. A study revealed that *R. dentatus* polyphenol-rich extract upregulated PPAR*γ*, and suppressed inflammation and oxidative stress [50]. Another study also showed that *R. dentatus* methanolic extract exerts anti-inflammatory effect at a dose of 500 mg/kg (*p.o*) in mice (Table 2) [16]. However, the exact compound responsible for such activities still awaits to be identified.

### 7.7. Other Biological Activities

According to Elsayed et al.'s findings, the aerial part of *R. dentatus* ethyl acetate extract displayed a powerful antidiabetic action in rats with type 2 diabetes. The administration of *R. dentatus* extract to diabetic rats resulted in a reduction in hyperglycemia, an improvement in glucose tolerance and insulin sensitivity, an increase in liver glycogen, and a reduction in the activity of enzymes involved in the metabolism of carbohydrates [50]. Another study reported that root extracts in methanol (LD_50_ = 867.80) and hexane (LD_50_ = 437) were effective in brine shrimp mortality assay at 1000 ppm [76, 78]. Furthermore, another study reported that the hot water root extract of *R. dentatus* exhibited molluscicidal activity against the snails (Table 2) [79].

## 8. Conclusion and Future Perspectives

The significance of *R. dentatus* as a pleiotropic and pharmacological agent against cancer, oxidative stress, inflammation, and neurodegeneration is outlined in this paper. Recent research conducted both in vitro and in vivo on a variety of biological systems has provided evidence that validates the pharmacological effects of the substance. *R. dentatus* is an essential component in the fight against cancer as well as inflammation, oxidative stress, and infections caused by bacteria and fungi. It is still necessary to conduct preclinical and clinical studies in order to fully understand or identify the potential of *R. dentatus* as a leading candidate for therapeutic agents in the treatment of a variety of chronic diseases, despite the fact that *R. dentatus* possesses good therapeutic potential.

## Figures and Tables

**Table 1:** Chemical compounds isolated from *Rumex dentatus*.

Classification	No	Chemical component	Part of plant	Reference
Quinones	1	Chrysophanol	Root	[40]
	2	Physcion		
	3	Emodin		
	4	Endocrocin		
	5	Chrysophanol-8-O-*β*-D-glucopyranoside		
	6	Physcion-8-O-*β*-D-glucopyranoside		
	7	Emodin-8-O-*β*-D-glucopyranoside		

Chromones	8	7-Hydroxy-2,5-dimethylchromone	Root	[40]
	9	2-Methyl-5-carboxymethyl-7-hydroxychromone		

Naphthalene glucoside	10	6-Methyl-7-acetyl-1, 8-dihydroxy-3-methoxy naphthalene-1-O-*β*-D(L)-glucoside	Root	[41]
	11	6-Methyl-7-acetyl-1, 8-dihydroxy naphthalene-1-O-*β*-D(L)-glucoside		

*C*-Glucosyl anthrones	12	Rumejaposide E	Root	[42]
	13	Rumejaposide F		
	14	Rumejaposide G		
	15	Rumejaposide H		
	16	Cassialoin		
	17	Rumejaposide I		

Flavonoids	18	Quercetin	Aerial part	[43]
	19	Rutin		
	20	Myricetin		[16]
	21	Kaempferol		
	22	Avicularin		[44]
	23	Quercitrin		
	24	Kaempferol 3-O-*β*-galactoside		[45]
	25	Kaempferol 3-O-*β*-glucoside		
	26	Kaempferol 3-O-rutinoside		
	27	Isorhamnetin 3-O-*β*-galactoside		
	28	Isorhamnetin 3-O-*β*-glucoside		
	29	Isorhamnetin 3-O-rutinoside		

Stilbenes	30	Resveratrol	Root, stem, leaf	[46]
	31	Polydatin	Root stem	

Monoterpenes	32	*α*-Thujene	Aerial part	[47]
	33	Limonene		
	34	Fenchone		
	35	Estragole		
	36	Anethole		

Fatty acid methyl esters	37	Meristic acid methyl ester	Aerial part	[47]
	38	Palmitic acid methyl ester		
	39	Stearic acid methyl ester		
	40	10-Oleic acid methyl ester		
	41	Linoleic acid		
	42	Linolenic acid methyl ester		
	43	C20:0 Methyl ester		
	44	Hydrocarbon C23H48		
	45	C22:0 Methyl ester		
	46	Squalene		
	47	Hydrocarbon C44H90		
	48	C24:0 Methyl ester		
	49	C26:0 Methyl ester		

Others	50	Helonioside A	Root	[48]
	51	Gallic acid		
	52	Isovanillic acid		
	53	*p*-Hydroxycinnamic acid		
	54	Succinic acid		
	55	*n*-Butyl-beta-D-fructopyranoside		
	56	Hexadecanoic acid 2, 3-dihydroxy propyl ester		
	57	Beta-sitosterol		
	58	Daucosterol	Aerial part	[48]
	59	Hexacosanoic acid		[49]
	60	Hexacosanol		[49]
	61	Vitamin C		[16]
	62	Chlorogenic acid		[16]
	63	*p*-Hydroxybenzoic acid	Leaves and root	[39]
	64	Syringic acid		
	65	Vanillin		
	66	Benzoic acid		
	67	Ferulic acid		
	68	Cinnamic acid		
	69	(S)- 4′-Methylnonyl benzoate	Aerial part	[50]

**Table 2:** Pharmacological effects of *R. dentatus*.

Pharmacological activity	Extract/compound	Detail	Minimum active concentration/dose	*In vitro*/*In vivo*	Reference
Anticancer Activity	Chrysophnol	Antiproliferative activity	IC_50_ values (*µ*M)	*In vitro*	[41]
		MCF-7	20.4 ± 7.8		
		7901	513 ± 265		
		A375	83.1 ± 35.1		
		SKOV-3	5.62 ± 1.58		
	6-Methyl-7-acetyl-1, 8-dihydroxy-3-methoxy naphthalene-1-O-*β*-D(L)-glucoside	MCF-7	269 ± 133		[41]
		A375	186 ± 57		
		SKOV-3	40.7 ± 23.1		
	6-Methyl-7-acetyl-1, 8-dihydroxy naphthalene-1-O-*β*-D(L)-glucoside	MCF-7	1580 ± 1860		[41]
		A375	275 ± 143		
		SKOV-3	174 ± 114		
	Methanol extract chloroform extract	Antiproliferative activity	IC_50_ values		[68]
		Breast cancer MDA-MB-231 cell line	111 *µ*g/mL		
			83 *µ*g/mL		
	Methanol extract	Cytotoxic and antitumor	250 µg/mL		[45]
		Ehrlich ascites carcinoma cell			

Antioxidant activity	Ethanol extract Methanol extract Petroleum ether extract Ethylacetate extract Chloroform extract Butanol extract	Scavenging activity against DPPH, hydroxyl, superoxide radicals, catalase, and lipid peroxidation	50−300 *µ*g/mL	*In vitro*	[38, 39, 75]

Antibacterial activity	70% Methanol extract	*B. megaterium*	5−25 mg/mL	Broth dilution/*In vitro*	[45]
		*B. subtilis*			
		*E. coli*			
		*S. aureus*			
		*K. pneumonia*			
		*Enterobacter*			
		*P. aeruginosa*			
	Ethanol extract Methanol extract Ethyl acetate extract Chloroform extract Butanol extract	*S. flexneri*	150−500 *µ*g/mL		[38, 75]
		*K. pneumonia*			
		*E. coli*			
		*P. aeruginosa*			
		*S. typhimurium*			
		*S. aureus*			
	Methanol extract	*S. aureus*	20 mg/mL		[76]
		*B. subtilis*			
		*M. luteus*			
		*E. coli*			
		*P. picketii*			
		*B. bronchiseptica*			

Antifungal	Ethanol extract Methanol extract Petroleum ether extract Ethylacetate extract Chloroform extract Butanol extract	*A. Versicolor* *A. flavus* *Acremonium* *P. dimorphosporum* *C. albicans* *C. krusei* *C. parapsilosis*	150−500 *µ*g/mL	Broth dilution/*In vitro*	[38, 75]
	Methanol extract Hexane extract	*F. solani* *A. flavus* *A. Niger* Mucor species *A. alterata* *A. fumigatus* *F. moniliforme*	12 mg/mL		[76]
Antiviral activity	Methanol extract Ethanol extract Benzene extract Chloroform extract Hexane extract	Dengue virus (DENV-2)	IC_50_ values (µg/mL) 0.154–0.499	*In vitro*	[77]

Others	Ethyl acetate extract	Antidiabetic activity	50 mg/kg Orally/4 weeks	*In vivo*	[50]
		↓ Insulin resistance			
		↓Hyperglycaemia			
		↓ Liver injury			
		↓ Oxidative stress			
		↑Carbohydrate metabolism			
		↑PPAR*γ*			
	Methanol extract Hexane extract	Cytotoxicity Brine shrimp	LC_50_ = 437–53970 ppm	*In vitro*	[76]
	Methanol extract		LC_50_ = 42 *µ*g/mL		[78]
	Hot water extract	Molluscicidal activity	0.2% (w/v)		[79]

## Data Availability

All data generated or analyzed during this study are included in this article.
